# Leveraging a digital network of pharmacy professionals to test a technology-assisted model to improve pharmacy access to quality-assured COVID-19 rapid antigen tests approved for self-use in Vietnam

**DOI:** 10.1371/journal.pone.0318331

**Published:** 2025-03-19

**Authors:** Tien Ho, Jaca Maison Lailo, Edrick Ramoran, Karishma Mutreja, Anabel Gomez, Michael Gallo, Josselyn Neukom

**Affiliations:** 1 Public Health Division, SwipeRx, Ho Chi Minh City, Vietnam; 2 Public Health Division, SwipeRx, Singapore, Singapore; 3 Market Access Division, FIND, Geneva, Switzerland; Ludwig Boltzmann Institute of Osteology, UKRAINE

## Abstract

Expanding access to rapid and self-use diagnostics in low-and-middle-income countries (LMICs) is crucial for advancing universal health coverage and creating more self-care opportunities. This study aimed to test the ability of a technology-assisted pharmacy activation model in Vietnam to improve access to quality-assured COVID-19 self-tests through the retail pharmacy channel. SwipeRx, a digital network with more than 30,000 pharmacy professionals in Vietnam, was leveraged to raise awareness about the benefits of COVID-19 rapid antigen tests (Ag-RDTs) that meet quality assurance standards for self-use and were registered with the Vietnamese regulatory authority. The campaigns also included information on local suppliers of *Humasis* and *Flowflex* self-test products, offering favorable pricing terms for SwipeRx users. In addition, offline pharmacy training and point-of-sale materials were provided to encourage dispensing, counseling, and restocking of at least one locally registered self-use Ag-RDT for COVID-19. After nine months of engagement between 2022 and 2023, a digital survey was conducted among 331 retail pharmacies to assess the impact on knowledge and practices related to COVID-19 self-tests. Pharmacy professionals who received SwipeRx training reported greater confidence and knowledge in stocking, dispensing, and counseling clients on the proper use of COVID-19 self-tests. These trained professionals were also more capable of distinguishing between COVID-19 Ag-RDTs approved for self-use and those approved for professional use. By the end of the nine months, 70 (92%) of trained pharmacy professionals stocked at least one quality-assured self-test for COVID-19, compared to just 38 (29%) of untrained professionals. These findings demonstrate that digital pharmacy networks can rapidly facilitate market introduction and access to new diagnostic products. Future initiatives should prioritize continuous price negotiations with manufacturers and distributors, focusing on optimizing pricing, credit, and delivery terms for community pharmacies. Additionally, combining digital and offline training with community demand-generating activities could facilitate greater pharmacy uptake of Ag-RDTs and other prioritized public health products.

## Introduction

Pharmacies are often the first or only point of healthcare access in low-and middle-income countries (LMICs) in Asia, such as Vietnam, especially for populations that are underserved by the public health system such as those in rural communities and low-income urban residents [1]. In many cases, community pharmacies are the closest and most accessible sources of health advice, medication and public health products including Ag-RDTs [2]. Due to their long operating hours, familiarity with clients, and quick service, retail pharmacies are a popular source of healthcare [1]. The COVID-19 pandemic amplified the role of community pharmacies, as pharmacies remained open across Southeast Asia when other healthcare providers became inaccessible [3]. Community pharmacies have great potential to help countries achieve universal health coverage (UHC) as a result of their role as primary healthcare providers and their ability to expand access to self-care including self-testing [4–6].

Enhanced access to quality assured Ag-RDTs, including self-tests, is crucial for rapid disease diagnosis and management, particularly in resource-limited settings [7]. Studies have shown that self-testing is both acceptable, effective, and feasible in LMICs for diseases such as COVID-19 [8], Hepatitis C [9], and HIV [10]. Empowering individuals with multiple diagnostic options, including quality assured self-use tests, can facilitate more timely access to confirmatory diagnosis and treatment, ultimately generating positive impacts on individual health outcomes as well as national disease burden and health systems [11,12].

Despite the fact that rapid diagnostics are critical tools in the fight against COVID-19 and other infectious diseases, access to quality-assured, affordable Ag-RDT in low and middle-income countries (LMICs) is sub-optimal [13]. For example, during the peak period of spread of the Omicron variant of COVID-19 (December 2021 – March 2022), the median testing rate for high-income countries was 600 tests/100,000 people/day, compared to 14 tests/100,000 people/day in LMICs [14]. Within the Ag-RDT category, access to quality-assured self-testing is further limited [15]. The COVID-19 pandemic highlighted the need for convenient access to point-of-care testing (POCT), including quality assured self-tests, given the need to shift screening from health facilities to communities. By facilitating self-testing, overburdened health systems are able to increase access for underserved communities at risk and strengthen the overall health system [16].

Broader systemic issues often limit access to diagnostics in LMICs. Regulatory barriers can delay the approval of new diagnostic tools, creating a time lag between development and deployment [17]. Additionally, supply chain inefficiencies, including poor infrastructure and distribution networks, hinder timely delivery of tests, especially beyond larger, urban health facilities [7]. Economic factors, including the high cost of testing, also restrict access [18].

While efforts to train and equip pharmacies with products and skills needed to contribute to public health priorities are far from new, to date most of these initiatives fail to achieve scale given the fragmented nature of the pharmacy channel [19]. Across Vietnam and other markets in southeast Asia, more than 80% of all retail pharmacies are independent [20]. As a result, the time and resources needed to engage the pharmacy channel to introduce new life-saving products or build pharmacist capacity to appropriately dispense and counsel remains prohibitive for most market development or public health initiatives. In this context, technology represents potential to improve scale, efficiency and value of pharmacy engagement efforts [21]. This is particularly relevant given the post-pandemic reality of increased digitization of pharmacy operations and health systems [22,23].

This study assessed the use of a pharmacy-focused digital platform, SwipeRx, to improve access to quality assured Ag-RDT self-tests for COVID-19 in Vietnam. SwipeRx is a free, all-in-one application that provides multiple features for pharmacy professionals and students across six markets in southeast Asia -- including accredited professional education courses, pharmaceutical news, peer and expert discussion forums, a drug directory, and other e-tools to improve pharmacy operations.

## Methods

During a 9-month pharmacy engagement period, SwipeRx utilized digital campaigns together with targeted pharmacy ‘activation’, which included in-pharmacy training and placement of client-facing point of sale materials (POSM), to motivate community pharmacies to stock quality assured self-tests for COVID-19 through local suppliers. No subsidy was provided, instead SwipeRx negotiated with suppliers for discounted commercial price and delivery terms to be offered to SwipeRx-app users who completed the training. This project built on an ongoing partnership with the Foundation for Innovative New Diagnostics (FIND), a global non-profit organization focused on promoting innovation in diagnostics, who served as a technical and financial partner throughout design, execution, and evaluation phases. Several monitoring indicators were tracked monthly, including the number of pharmacies activated by SwipeRx, the number of self-use Ag-RDTs for COVID-19 purchased by activated pharmacies, and exposure and engagement metrics for the digital campaign, using an activation dashboard designed in October 2022. The pharmacy-engagement period was followed by a digital survey designed to assess differences in COVID-19 Ag-RDT knowledge and practices between SwipeRx-activated and non-activated pharmacies.

### Digital campaign modes and messaging

With inputs from FIND, the Ministry of Health and pharmacists using SwipeRx in Vietnam, SwipeRx developed digital campaigns to promote the benefits of two locally registered Ag-RDT for COVID-19 that met standards for quality assured self-testing to pharmacy professionals using SwipeRx across Vietnam. This approach included a combination of digital posters and interactive games designed to promote the benefits of stocking and recommending use of Flowflex and Humasis 5-unit packages of self-use Ag-RDT for COVID-19. Both of these tests were approved by Vietnam’s Ministry of Health’s Department of Medical Equipment and Construction, with Humasis approved on 20 August 2021 and Flowflex approved on 1 November 2021.

Round 1 campaign messages promoted these two brands of nasal Ag-RDTs for COVID-19 as (1) easier to use compared to nasopharyngeal Ag-RDTs due to a shorter nasal swab; (2) designed for self-use outside of health-facilities given product design and packaging; and (3) available for pharmacy purchase from local commercial suppliers offering SwipeRx users commercial prices discounted by up to 60%. The first round of the campaign—including 12 posters and 6 interactive games--was deployed through the SwipeRx newsfeed and social media channels during October-November 2022.

In response to pharmacist feedback and changes in the market context, the campaign was revised in January 2023. Round 2 of the digital campaign included 14 posters and 6 games which were deployed in February-March 2023. The updated digital campaign materials emphasized affordability and quality of the quality assured self-test brands and explained that Ag-RDT products locally advertised as integrated tests for flu as well as COVID-19 were neither registered with the Vietnamese regulatory authority nor globally recognized as meeting quality assurance standards.

### In-pharmacy activation: training, coaching & POSM placement

To complement the digital campaign, in Ha Noi, Ho Chi Minh City and Quang Binh province, SwipeRx collaborated with local suppliers to integrate in-pharmacy training, post-training coaching and placement of client-facing point-of-sale-material (POSM) to prompt stocking, counseling and sales at community pharmacies. These pharmacy-engagement activities are collectively referred to as ‘activation’. A team of 2 SwipeRx pharmacists with sales experience (based in Ho Chi Minh City) and 4 medical representatives working for local pharmaceutical distributors (3 based in Ha Noi and 1 based in Quang Binh province) were equipped with digital training and post-training coaching tools to motivate retail pharmacies to stock and re-stock quality assured self-tests for COVID-19. The focus locations for pharmacy activation were selected considering i) resources and time available; ii) higher COVID-19 burden in the two main urban centers; and iii) interest in assessing results in at least one province outside of the two main urban centers of the country.

Pre-and post-training knowledge levels were measured among pharmacists trained by the SwipeRx team using ten standardized questions linked to training objectives. Following the initial onsite training and decision to stock at least 1 of the locally registered quality assured self-test products (Humasis or Flowflex 5-test packages), trained pharmacies received client-facing POSM (countertop wobblers and stickers) to trigger purchase and counseling for appropriate use of the quality assured self-tests. Trained pharmacies received monthly coaching through the “Zalo” mobile messaging service as well as in-pharmacy sessions. Local commercial suppliers were notified of self-use Ag-RDT for COVID-19 orders facilitated by SwipeRx, and deliveries were made within 1-2 days depending on pharmacy location.

### Survey design, inclusion criteria, and sample size

The primary research method for this study was a quantitative, digital survey deployed through the SwipeRx app. The survey objectives were to: (1) assess pharmacy availability and affordability of COVID-19 Ag-RDTs, including quality assured self-tests (2) assess preferences and factors influencing pharmacy stocking practices of COVID-19 Ag-RDTs (3) identify enablers and barriers to improving pharmacy access to quality assured self-use Ag-RDTs; and (4) assess knowledge and attitudes among pharmacy professionals regarding quality assured, self-use tests by SwipeRx-activation status. The digital survey targeted non-student pharmacy professionals (defined specifically as pharmacists, pharmacy assistants, managers, and owners working in retail pharmacies) who were active SwipeRx app users and 18 years or older. Recruitment strategies included app push notifications, SMS, phone calls, key opinion leader outreach, social media, and pharmacy visits. The survey aimed to collect responses from 200 eligible retail pharmacy professionals, of which at least 35% were to be from a rural district. This sample size calculation was based on the following formula [24,25], where N was the population of pharmacy professionals registered on SwipeRx in Vietnam at the time of the study. The sample proportion (p) was set at 0.5 to yield a conservative sample size. The margin of error was set at 5% and the Z statistic was set at 1.96 for a confidence level of 95%.


$$n=\frac{N*X}{\left(X+N-1\right)}whereX=\frac{1.96*p*\left(1-p\right)}{{\left(marginoferror\right)}^{2}}$$

Between 50-100 respondents were targeted from activated pharmacies with at least one professional who received training, coaching and POSM-placement through the SwipeRx activation efforts.

### Survey development and distribution

Following approval of the survey questionnaire and protocol by the Ha Noi University of Public Health (see details below), the English questionnaire was translated to Vietnamese and pre-tested with 5 Vietnamese-speaking SwipeRx users to ensure access, time requirements, and understandability/acceptability of questions. A bilingual, Vietnamese pharmacist was responsible for translating the final English research questionnaire into Vietnamese. Independent back-translation was arranged by another bilingual SwipeRx team member who had no prior exposure to the original English version. Discrepancies identified through the comparison of the back-translated and original English versions were addressed before the research questionnaire was finalized in Vietnamese. Following minor revisions to the questionnaire identified during pre-testing, the final survey questionnaire included 38 questions. Among the total questions, 20 were mandatory for respondents stocking COVID-19 self-tests and 12 questions were mandatory for respondents not stocking any COVID-19 self-tests. The survey included questions related to respondent demographics, COVID-19 self-test stocking practices, pharmacy client profile/volume, knowledge and training experience related to COVID-19 self-tests, attitudes towards COVID-19 self-tests, and perceived opportunities and barriers to pharmacy access and uptake/use of COVID-19 self-tests (Supplementary File 1: Survey Questionnaire). Respondents could withdraw from the digital survey at any time. The survey was hosted on the Qualtrics platform (http://www.qualtrics.com; Qualtrics, Utah, USA) and was accessed through a digital invitation containing a weblink to the site through SwipeRx. Pharmacy professionals who followed the link were asked to provide informed consent prior to completing the survey online. Those who did not provide consent could not access the survey questions. Access to the survey was allowed only once for each SwipeRx user identification code or mobile number to minimize the risk of duplication.

### Ethics approval & analysis of survey results

Ethics approval for the study was obtained from the Hanoi University of Public Health prior to data collection (approval number 023-312 received 6 June 2023). All survey respondents provided informed consent. The survey was accessible through the SwipeRx platform between 20 June and 18 July 2023. Pharmacy professionals were recruited to complete the online survey via in-app pushes, SMS, phone calls, key opinion leader (KOL) promotion, SwipeRx social media page posts, and face-to-face by SwipeRx and affiliated commercial sales agents in the three focus cities/provinces, who integrated promotion of the survey into their ongoing coaching visits. Survey respondents who completed at least 80% of the survey questions received mobile phone credit equivalent to 6 US dollars. To maintain anonymity, those eligible for the incentive were only asked to share the telephone numbers that they wished the credit to be transferred to. Each respondent was recorded by their unique numeric SwipeRx identification code. No personal identifiable information was collected through the survey.

Data collection, cleaning and analysis was performed by members of the SwipeRx research and public health team. Results were exported from Qualtrics into Google Sheets and any necessary case duplication removal was performed (verified by the unique SwipeRx ID and mobile number). Only responses that were 80% complete were included in the analyses. Data were then analyzed using STATA 16 software (StateCorp, College Station, TX, USA). Data are presented as medians for continuous responses and as percentages/percentage distribution for categorical responses. Additionally, percentages reported are calculated based on respondents who answered a specific question. Data were analyzed using a variety of techniques depending on the nature of data and sampling including: (1) descriptive analysis of demographics, client volume, knowledge, and practices; (2) bivariate analysis of differences by SwipeRx-activation status and other indicators. Analysis of test information included participants who provided complete information only. Only findings which had statistical significance at the 95% level of significance are included in this manuscript.

## Results

This study aimed to test a technology-assisted pharmacy activation model to improve knowledge about and practices related to the provision of quality-assured self-tests for COVID-19 through community pharmacies in Vietnam.

### Digital campaign coverage & engagement

Cumulatively, 360,694 SwipeRx user and social media account exposure points across Vietnam were generated by digital campaign content designed to emphasize the benefits of quality assured COVID-19 self-tests. Round 2 exposure and engagement (defined as likes, comments or shares) results increased compared with Round 1 as a result of greater use of interactive content and digital promotion techniques. Round 1 exposure points totaled 95,667, while Round 2 generated 265,027 exposure points. Round 1 engagement points totaled 5,353, falling short of the target of 10,000. However, the second round of deployment achieved 16,853 points, exceeding the target of 10,000. Increased exposure and engagement results during the second round were achieved by increasing interactive content and calls-to-action (for engagement) as well as increasing digital promotion strategies through the SwipeRx app and social media channels.

### Training, coaching & POSM placement: supply chain activation

**Fig 1:**
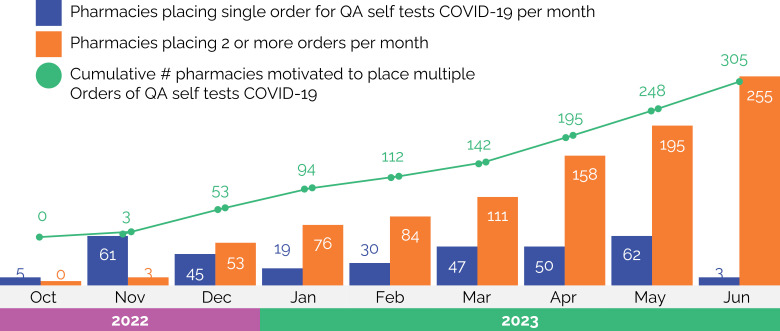
Number of pharmacies motivated to stock quality assured self-use Ag-RDT by month.

During the 9-month activation period, 331 pharmacies including 144 (43.4%) in Ho Chi Minh, 146 (44.2%) in Ha Noi (44.2%), and 41 (12.4%) in Quang Binh were activated as a result of completing the SwipeRx training, procuring 17,115 quality assured COVID-19 self-tests from commercial suppliers including 11,467 (67%) Flowflex brand and 5,648 (33%) Humasis brand. This result exceeded the target of activating 100 pharmacies through sales of 11,250 self-tests. Among the 331 activated pharmacies, 305 (92%) reordered additional supplies of quality assured self-tests for COVID-19 at least once during the 9-month period. There were 10 pharmacies that stocked quality assured tests but did not agree to complete the training, including the post-training questionnaire. Comparison of post vs pre training knowledge levels among pharmacy professionals trained revealed improved knowledge about the specific attributes and benefits of quality assured self-tests for COVID-19 (Fig 1).

### Survey respondent characteristics

In total, 210 pharmacy professionals from Vietnam completed the digital survey (Table 1), exceeding the target of 200 respondents. 123 respondents came from urban districts (59%). There were 160 (76%) females, and 111 (53%) were pharmacists. Out of the pharmacists surveyed, 77 (37%) had been activated with SwipeRx training, coaching and POSM placement, while 133 (63%) were not activated by SwipeRx.

**Table 1:** Respondent characteristics.

	Vietnam (N = 210)
Provinces covered, n/N	27/63
Female, n (%)	160 (76)
Average age (years)	33
Setting, n (%)	
Urban	123 (59)
Rural	87 (41)
Profession, n (%)	
Pharmacist	111 (53)
Pharmacy assistant	16 (8)
Pharmacy owner	68 (32)
Pharmacy manager	15 (7)
SwipeRx Activation Status, n (%)	
Trained, Coached & POSM Placed	77 (37)
Non-trained	133 (63)
Currently Stocking COVID-19 Ag-RDT (any type), n (%)	
Yes	184 (88)
No	26 (12)

### Pharmacy clientele and Ag-RDT purchasing

On average, survey respondents reported 50 clients (Interquartile Range: 35 to 70 clients) visiting their pharmacy each day. In the activation locations, respondents from Ho Chi Minh reported 50, Hanoi 48, and Quang Binh 60 pharmacy client visits in each day. Among all survey respondents, 95 (45%) reported that clients purchase COVID-19 self-tests less frequently than once every other month. Survey respondents reported that the most common client type purchasing Ag-RDT (any type) were individual clients with COVID-19 symptoms (reported by 189 (90%) respondents), followed by senior citizens (reported by 55 (26%) respondents), health workers (reported by 44 (21%) respondents), business owners (reported by 22 (10%) respondents), and others (reported by 13 (6%) respondents). Common COVID-19 self-test related questions from pharmacy clients reported by survey respondents related to self-testing methods, differences between test types, pricing, and the country of origin where tests were manufactured.

### Pharmacy services related to COVID-19

184 (88%) survey respondents reported providing COVID-19 related counseling at their pharmacy, whereas 130 (62%) reported referring clients needing COVID-19 services to a health facility and 89 (43%) reported offering in-pharmacy testing. Respondents from pharmacies activated through SwipeRx training, coaching and POSM placement were more likely to report counseling (reported by 75 (97%) respondents) and providing in-pharmacy COVID-19 self-test (reported by 40 (52%) respondents) and more likely to report referring clients to a health facility (reported by 41(53%) respondents) compared to non-trained respondents (Table 2).

**Table 2:** Pharmacy activities related to COVID-19 by type and activation status.

Trained by SwipeRx n (% column)	Non-Trained by SwipeRx n (% column)	Reported Pharmacy Practices Related to COVID-19
40 (52%)	49 (37%)	Conduct in-pharmacy COVID-19 testing
41 (53%)	89 (67%)	Refer clients for COVID-19 to a health facility
75 (97%)	109 (82%)	Counsel clients related to COVID-19

### COVID-19 self-test stocking & pricing

109 (52%) survey respondents reported stocking at least 1 of either Flowflex or Humasis Nasal Ag-RDTs. Pharmacy professionals activated by SwipeRx training and coaching were more likely (reported by 70 (92%) respondents) to stock at least 1 of the Flowflex or Humasis Nasal self-tests compared to non-trained professionals (reported by 38 (29%) respondents). In total, 19 different self-tests were reported as being stocked at retail pharmacies in Vietnam as of in July 2023, although 6 brands were reported by only 1 pharmacy. All tests were locally approved, but only 9 of 19 met global quality assurance requirements as determined by a WHO listed Stringent Regulatory Authority. Compared to the less frequently stocked brands at pharmacies, the average (trade) purchase price of Flowflex and Humasis Nasal Ag-RDTs was lower. For example, of the 16 COVID-19 self-tests that were stocked by less than 21(10%) respondents, the average reported pharmacy purchase price per stock-keeping unit (SKU) of Ag-RDTs for COVID-19 was $17.32, whereas the average trade purchase price reported by pharmacists for a box of 5 Flowflex nasal tests was $4.87 and the average purchase price reported by pharmacists for a box of 5 Humasis nasal tests was $7.67.

Factors reported as influencing pharmacy COVID-19 Ag-RDT stocking decisions included trade (pharmacy purchase) price, consumer preference and discounts or other deals from suppliers. 56 (31%) survey respondents reported global approvals and 52 (28%) reported local approvals as influencing pharmacy stocking decisions. Rural respondents were more likely to report consumer preferences as influencing stocking (χ^2^ =  18.89 p =  0.001) whereas urban respondents were more likely to report global approvals (χ^2^ =  6.24 p =  0.012), information from SwipeRx (χ^2^ =  6.15 p =  0.003), and local approvals (χ^2^ =  4.22 p =  0.040).

### COVID-19 Ag-RDT knowledge, attitudes & motivations

158 (75%) survey respondents were able to correctly identify differences between COVID-19 Ag-RDTs approved for self-use compared to professional use. This was higher among urban respondents (reported by 104 (85%) respondents) compared to rural (reported by 54(62%) respondents) (χ^2^ =  13.83 p =  0.001). SwipeRx activated respondents were also more likely to be able to identify the difference between Ag-RDT approved for self-use and Ag-RDT approved for professional use (Table 3).

**Table 3:** Key findings: Ability to Identify Self-Use Ag-RDT & Confidence in COVID-19 counseling.

Activated by SwipeRx; n = 77 *n (%col)*	Non-activated; n = 133 *n (%col)*	Finding
**70 (91%)** ^*^	88 (66%)	Able to correctly identify differences between COVID-19 Ag-RDT approved for self-use vs COVID-19 Ag-RDT approved for professional use
**“Very Confident”** ^*^	“Confident”	In counseling ability related to COVID-19

* denotes statistical significance at 5% level.

81 (39%) survey respondents strongly agreed that pharmacists have a role to play in counseling and referring self-test clients from their pharmacy and 197 (95%) reported they were confident or very confident in their counseling ability. SwipeRx activated respondents were more likely to report confidence in their ability to counsel clients about COVID-19 compared to respondents who were not trained by SwipeRx (Table 2).

When asked to identify factors related to quality of Ag-RDT, 52 (68%)of sampled pharmacy professionals identified global approvals and 61 (79%) identified local approvals. In addition, 132 (63%) respondents identified location where the test was manufactured and price as factors related to test quality. Rural respondents were more likely to report manufacturing location (reported by 64 (74%) respondents) and local approvals (reported by 67 (77%) respondents) as factors perceived relating to quality compared to urban respondents (χ^2^ =  7.29 p =  0.007; χ^2^ =  4.41 p =  0.036). Respondents activated by SwipeRx were more likely to identify local approvals and packaging as key to quality of Ag-RDT compared to non-trained respondents (Table 4).

**Table 4:** Key findings: perceived aspects of Ag-RDT quality.

Activated by SwipeRx; n = 77 *n (%col)*	Non-activated; n = 133 *n (%col)*	Quality Perceptions
**52 (68%)** ^*^	111 (83%)^*^	Believe “quality” of Ag-RDT relates to global approvals
**61 (79%)** ^*^	84 (63%)^*^	Believe “quality” of Ag-RDT relates to local approvals
**46 (60%)** ^*^	60 (45%)^*^	Believe “quality” of Ag-RDT relates to packaging

* denote statistical significance at 5% level.

Discounts (reported by 156 (75%) respondents), free product promotions (reported by 131 (63%) respondents) or pharmacy equipment (reported by 126 (60%) respondents) and credit terms (reported by 104 (50%) respondents) were identified as having potential to motivate pharmacies to stock quality assured COVID-19 tests by survey respondents (n = 209.) Respondents activated by SwipeRx were more likely to identify price reductions and MOH endorsement as factors that could increase pharmacy sales of quality assured self-tests. Non-trained pharmacy professionals were more likely to identify in-pharmacy materials as influential. Activated respondents were more likely to express interest in participating in future programs related to quality Ag-RDT designed for self-use (reported by 76 (99%) respondents) compared to non-trained (reported by 116 (89%) respondents).

## Discussion

Throughout the COVID-19 pandemic, the WHO recommended the prioritizing of access to a variety of self-care interventions, including health products and rapid tests accessed through over-the-counter at pharmacies and drugstores [26]. Consistent with this recommendation, SwipeRx used a combination of digital and offline techniques to improve availability and affordability of quality assured self-tests (Humasis or Flowflex), at community pharmacies in 2 cities and 1 province of Vietnam. Pharmacy professionals activated by SwipeRx were three times more likely to stock quality assured self-tests compared to non-activated pharmacies. Additionally, pharmacy professionals who were engaged by SwipeRx through digital campaigns and offline training, coaching and POSM placement were more likely to report confidence in counseling clients about self-tests and other aspects of COVID-19. These pharmacy professionals were also more knowledgeable about the difference between quality assured tests approved for self-use compared to professional use. Guidance from the WHO emphasizes the importance of policy support for evidence-based, quality self-care options and health worker training to promote self-care options [27]. Results described in this paper are important in the context of WHO and other global guidance, as well as the logistical challenges associated with scaling pharmacy engagement in LMICs given the fragmentation of this channel [28]. Although self-care interventions cannot overcome all gaps in under resourced health systems, enhancing access to tools for self-care can support greater health coverage and advance the right to health for all [27].

Close to one-third--60 (29%) of all pharmacy professionals surveyed reported at least 1 stock-out in Ag-RDT for COVID-19 in the month prior to June 2023 when data was collected. Stock-outs were higher among rural respondents, who face more prominent supply chain challenges, highlighting the need to continue to strengthen the pharmacy supply chain for Ag-RDTs and other public health products. The finding that most pharmacists reported sourcing Ag-RDT for COVID-19 from multiple suppliers in a given month is consistent with other research and highlights the need for consistent access to quality assured Ag-RDT at competitive prices at retail pharmacies [29,30]. Resolving bottlenecks to ensure continuous supply of quality assured, affordable Ag-RDT through local commercial suppliers is essential to ongoing efforts to expand pharmacy contributions to point-of-care testing for COVID-19 and other public health priorities [31].

Findings from this research also emphasize the importance of Ag-RDT pricing for both pharmacy professionals and their customers. Flowflex and Humasis were the most highly stocked Ag-RDTs and had lower averages prices compared to the less frequently reported brands stocked at pharmacies. Other studies have shown a correlation between subsidy for self-tests and uptake of self-tests [32]. The study describes results achieved without subsidy. Instead, negotiations with suppliers for discounted commercial pricing and delivery terms offered to SwipeRx-affiliated pharmacies generated improvements in Ag-RDT affordability and access through SwipeRx-community pharmacies. In the future, similar pharmacy engagement programs should negotiate with manufacturers at global and regional levels – as well as with in-market distribution partners--to include volume or time-based discounts and credit and delivery terms to optimize access gains. Given the finding that Ag-RDT availability (as well as other access parameters including supply chain efficiency and pharmacy professionals’ knowledge) was lower at sampled rural pharmacies, future activations should include a purposeful focus on engaging rural pharmacies. Price (to the trade) as well as discounts, incentives, credit terms were all described as influencing pharmacy stocking practices related to COVID-19 Ag-RDT by more than half of survey respondents. The negotiated price reduction for Flowflex self-tests (from 30,000 VND in October-December 2022 to 12,000 VND from January-June 2023) contributed to increases in pharmacy stocking and restocking during the last 6 months of the activation period. These findings highlight trade price as well as manufacturing location as key factors influencing sales. Perceptions regarding quality also matter, and manufacturer location as well as local and global approvals were emphasized as valued indicators of Ag-RDT quality by pharmacy professionals sampled in Vietnam.

Positive correlation between SwipeRx digital and offline activation and increases in pharmacy access to quality assured self-use Ag-RDT indicates potential for scaled use of SwipeRx to facilitate market introduction and access to Ag-RDTs and other public health products through the pharmacy channel in Vietnam and similar markets. Other studies have likewise demonstrated the potential impact of trade/health worker-focused, access and demand generating activities, such as interventions involving a field-based sales force that increased visits to private healthcare workers for tuberculosis care [33]. Previously published research also highlights the importance of supply chain strengthening measures, including negotiation with suppliers to improve availability and affordability of appropriately packaged POC diagnostic supplies [34]. The SwipeRx pharmacy activation experience highlighted the importance of early and ongoing negotiations with supply chain actors (manufacturers, importers, distributors and wholesalers) to ensure pricing and other terms are tailored to local market conditions, particularly for pandemic-related products given the variation in consumer demand and supplies as the rate of infections evolves over time. Regular communication with local suppliers about pricing and other terms for retail pharmacies is recommended, to ensure alignment with changes in local market conditions.

There were a range of contextual factors that may have affected results. Monthly reported COVID-19 caseload varied throughout the activation phase, dropping below 500 during February and March 2023, and rising to over 50,000 in May 2023. Pharmacy customer demand for COVID-19 tests declined during the 9-month activation period due to a drop in the reported caseload of COVID-19 across time, as indicated by the average number of Ag-RDT (any brand/type) sold by sampled pharmacies. The lack of complementary investment in community-demand generation may have limited results. Available Humasis nasal 5-test packs in Vietnam expired in July 2023 and the local distributor did not import additional supplies (with a later expiration date) during the activation period, which reduced trade willingness to stock this brand during the last 2 months of the activation. The local distributor of Flowflex tests was initially unwilling to offer discounts for SwipeRx users until January 2023 when they offered a substantial reduction in price from 30K to 12K per test. Corruption findings linked to public procurement of locally manufactured COVID-19 test kits in 2022 and subsequent investigations were widely covered media stories during the activation period [35]. Media coverage of the corruption and changes in COVID-19 case finding were factors that may have influenced perceptions of the importance of local and global approvals for Ag-RDT.

Several limitations warrant consideration when interpreting the results of this study. Firstly, due to the purposive nature of the sample, the findings cannot be generalized to all pharmacy professionals registered on SwipeRx or all pharmacy professionals working in Vietnam. Secondly, potential self-reporting biases could have led to discrepancies between the reported and actual pharmacy practices. Due to the presence of outliers and missing or incomplete data, the aggregated data may not accurately reflect the full context at all sampled pharmacies. Additionally, the potential for self-selection bias should be considered, as pharmacy professionals with a particular interest in COVID-19 or rapid diagnostics may have been more likely to complete the digital survey. Finally, with the activation and survey sample focused on two main cities and Quang Binh province, geographic limitations also need to be noted.

## Conclusion

By leveraging a digital network used by more than half of all retail pharmacists registered in Vietnam, it is possible to facilitate market introduction and access to new rapid diagnostic tests within a relatively short amount of time. Digital networks of pharmacy professionals can be leveraged to raise pharmacy professionals’ awareness of the benefits of quality assured self-tests and facilitate stocking of new health products through local commercial distribution networks. A combination of digital engagement, offline training, mobile and in-coaching and placement client-facing, in-pharmacy materials can be used to enhance pharmacy professionals’ confidence related to stocking, dispensing and counseling clients about appropriate self-use of quality assured Ag-RDTs.

These findings further demonstrate the importance of continuous negotiations with commercial manufacturers as well as their importer and distributor partners to ensure trade pricing and delivery terms for quality assured products are optimized. Future market shaping initiatives should include a focus on price discounts, credit and delivery terms targeting retail community pharmacies, ensuring adequate supplies of products with an expiration date that is greater than six months. While not part of the activation conducted by SwipeRx, investment in complementary, community demand-generating activities is likely to complement pharmacist capacity and supply chain strengthening investments to generate greater pharmacy uptake of quality assured Ag-RDTs and other prioritized public health products.

## Supporting information

S1 File SwipeRx Viet Nam: Understanding access to quality-assured COVID-19 Ag-RDTs for self-testing survey. (PDF)

## References

[1] Miller R, Goodman C. Performance of retail pharmacies in low- and middle-income Asian settings: a systematic review. Health Policy Plan. 2016;31(7):940–53. doi: 10.1093/heapol/czw007 26962123 PMC4977427

[2] Duggan C. Advancing the workforce to meet the Primary Health Care Agenda: pharmacy’s contribution to universal health coverage. Int J Pharm Pract. 2020;28(2):118–20. doi: 10.1111/ijpp.12579 32176414

[3] Querequincia JMB, Faller EM. A mini review on the community pharmacy practice experiences in selected Southeast Asian countries. GSC Biol Pharm Sci. 2023;23(2):129–32. doi: 10.30574/gscbps.2023.23.2.0193

[4] Kalita A, Bose B, Woskie L, Haakenstad A, Cooper JE, Yip W. Private pharmacies as healthcare providers in Odisha, India: analysis and implications for universal health coverage. BMJ Glob Health. 2023;8(Suppl 5):e008903. doi: 10.1136/bmjgh-2022-008903 37778756 PMC10546140

[5] Hedima EW, Okoro RN. Primary health care roles of community pharmacists in low- and middle-income countries: a protocol for a mixed methods systematic review. J Am Pharm Assoc (2003). 2023;63(5):1448–51. doi: 10.1016/j.japh.2023.06.011 37336265

[6] Alenezi S, Alanazi M, Aljazaeri R, Almuzaini M, Alrasheidi S, Shamlan WB, et al. Community pharmacies in the Asian countries of developing health system: formation, regulation, and implication. Pharmacy (Basel). 2023;11(4):127. doi: 10.3390/pharmacy11040127 37624082 PMC10460015

[7] Kuupiel D, Bawontuo V, Mashamba-Thompson TP. Improving the accessibility and efficiency of point-of-care diagnostics services in low- and middle-income countries: lean and agile supply chain management. Diagnostics (Basel). 2017;7(4):58. doi: 10.3390/diagnostics7040058 29186013 PMC5745394

[8] Martínez-Pérez GZ, Shilton S, Saruê M, Cesario H, Banerji A, Batheja D, et al. Self-testing for SARS-CoV-2 in São Paulo, Brazil: results of a population-based values and attitudes survey. BMC Infect Dis. 2022;22(1):720. doi: 10.1186/s12879-022-07706-7 36056299 PMC9438865

[9] Nguyen LT, Nguyen VTT, Le Ai KA, Truong MB, Tran TTM, Jamil MS, et al. Acceptability and usability of HCV self-testing in high risk populations in Vietnam. Diagnostics (Basel). 2021;11(2):377. doi: 10.3390/diagnostics11020377 33672241 PMC7926709

[10] Rivera AS, Hernandez R, Mag-Usara R, Sy KN, Ulitin AR, O’Dwyer LC, et al. Implementation outcomes of HIV self-testing in low- and middle- income countries: a scoping review. PLoS One. 2021;16(5):e0250434. doi: 10.1371/journal.pone.0250434 33939722 PMC8092786

[11] Wang C, Zhao P, Weideman AM, Xu W, Ong JJ, Jamil MS, et al. Expanding hepatitis C virus test uptake using self-testing among men who have sex with men in China: two parallel randomized controlled trials. BMC Med. 2023;21(1):279. doi: 10.1186/s12916-023-02981-w 37507702 PMC10386771

[12] Peeling RW, Boeras DI, Marinucci F, Easterbrook P. The future of viral hepatitis testing: innovations in testing technologies and approaches. BMC Infect Dis. 2017;17(Suppl 1):699. doi: 10.1186/s12879-017-2775-0 29143676 PMC5688478

[13] Chamas C, Barbeitas MM, Correa M, Kameda K, de Oliveira ACD, Villarinho L. Innovation in diagnostics: addressing gaps in low- and middle-income countries. Bull World Health Organ. 2022;100(8):467-467A. doi: 10.2471/BLT.22.288313 35923283 PMC9306385

[14] Han AX, Girdwood SJ, Khan S, Sacks JA, Toporowski A, Huq N, et al. Strategies for using antigen rapid diagnostic tests to reduce transmission of severe acute respiratory syndrome coronavirus 2 in low- and middle-income countries: a mathematical modelling study applied to Zambia. Clin Infect Dis. 2023;76(4):620–30. doi: 10.1093/cid/ciac814 36208211 PMC9619661

[15] Katoba J, Kuupiel D, Mashamba-Thompson TP. Toward improving accessibility of point-of-care diagnostic services for maternal and child health in low- and middle-income countries. Point Care. 2019;18(1):17–25. doi: 10.1097/POC.0000000000000180 30886544 PMC6407818

[16] Chan JTN, Nguyen V, Tran TN, Nguyen NV, Do NTT, van Doorn HR, et al. Point-of-care testing in private pharmacy and drug retail settings: a narrative review. BMC Infect Dis. 2023;23(1):551. doi: 10.1186/s12879-023-08480-w 37612636 PMC10463283

[17] Mugambi ML, Peter T, F Martins S, Giachetti C. How to implement new diagnostic products in low-resource settings: an end-to-end framework. BMJ Glob Health. 2018;3(6):e000914. doi: 10.1136/bmjgh-2018-000914 30498586 PMC6254739

[18] Hansen MA, Lekodeba NA, Chevalier JM, Ockhuisen T, Del Rey-Puech P, Marban-Castro E, et al. Cost of SARS-CoV-2 self-test distribution programmes by different modalities: a micro-costing study in five countries (Brazil, Georgia, Malaysia, Ethiopia and the Philippines). BMJ Open. 2024;14(4):e078852. doi: 10.1136/bmjopen-2023-078852 38631825 PMC11029185

[19] Thornewill J, Antimisiaris D, Ezekekwu E, Esterhay R. Transformational strategies for optimizing use of medications and related therapies through us pharmacists and pharmacies: Findings from a national study. J Am Pharm Assoc. 2022;62(2):450–60. doi: 10.1016/j.japh.2021.10.018 PMC8572696 34758925

[20] Vũ X. The growth of the pharmaceutical retail chain in Vietnam. Vietdata Research. 2023. [cited 2024 Sep 25] Available from: https://www.vietdata.vn/post/the-growth-of-the-pharmaceutical-retail-chain-in-vietnam

[21] Tran PMT, Dam TA, Huynh HB, Codlin AJ, Forse RJ, Dang HMT, et al. Evaluating novel engagement mechanisms, yields and acceptability of tuberculosis screening at retail pharmacies in Ho Chi Minh City, Viet Nam. PLOS Glob Public Health. 2022;2(10):e0000257. doi: 10.1371/journal.pgph.0000257 36962503 PMC10021543

[22] Golinelli D, Boetto E, Carullo G, Nuzzolese AG, Landini MP, Fantini MP. Adoption of digital technologies in health care during the COVID-19 pandemic: systematic review of early scientific literature. J Med Internet Res. 2020;22(11):e22280. doi: 10.2196/22280 33079693 PMC7652596

[23] Barata J, Maia F, Mascarenhas A. Digital transformation of the mobile connected pharmacy: a first step toward community pharmacy 5.0. Inform Health Soc Care. 2022;47(4):347–60. doi: 10.1080/17538157.2021.2005603 34855578

[24] Qualtrics. Sample Size Calculator. In: Qualtrics [Internet]. 2023. [cited 2024 Nov 13]. Available from: https://www.qualtrics.com/blog/calculating-sample-size/.

[25] Bruvold NT, Murphy RA. Sample sizes for comparison of proportions. Technometrics. 1978;20(4):437–40. doi: 10.1080/00401706.1978.10489698

[26] WHO. Maintaining essential health services: operational guidance for the COVID-19 context. Geneva: World Health Organization; 2020. Available from: https://iris.who.int/bitstream/handle/10665/332240/WHO-2019-nCoV-essential_health_services-2020.2-eng.pdf?sequence=1

[27] Narasimhan M, Karna P, Ojo O, Perera D, Gilmore K. Self-care interventions and universal health coverage. Bull World Health Organ. 2024;102(2):140–2. doi: 10.2471/BLT.23.290927 38313150 PMC10835638

[28] Smith F. The quality of private pharmacy services in low and middle-income countries: a systematic review. Pharm World Sci. 2009;31(3):351–61. doi: 10.1007/s11096-009-9294-z 19343530

[29] Poyer S, Shewchuk T, Tougher S, Ye Y, ACTwatch Group, Mann AG, et al. Availability and price of malaria rapid diagnostic tests in the public and private health sectors in 2011: results from 10 nationally representative cross-sectional retail surveys. Trop Med Int Health. 2015;20(6):744–56. doi: 10.1111/tmi.12491 25728761

[30] Cohen J, Fink G, Berg K, Aber F, Jordan M, Maloney K, et al. Feasibility of distributing rapid diagnostic tests for malaria in the retail sector: evidence from an implementation study in Uganda. PLoS One. 2012;7(11):e48296. doi: 10.1371/journal.pone.0048296 23152766 PMC3495947

[31] Kuupiel D, Tlou B, Bawontuo V, Drain PK, Mashamba-Thompson TP. Poor supply chain management and stock-outs of point-of-care diagnostic tests in Upper East Region’s primary healthcare clinics, Ghana. PLoS One. 2019;14(2):e0211498. doi: 10.1371/journal.pone.0211498 30811407 PMC6392218

[32] Mugo PM, Micheni M, Shangala J, Hussein MH, Graham SM, Rinke de Wit TF, et al. Uptake and acceptability of oral HIV self-testing among community pharmacy clients in Kenya: a feasibility study. PLoS One. 2017;12(1):e0170868. doi: 10.1371/journal.pone.0170868 28125699 PMC5268447

[33] Deo S, Jindal P, Sabharwal M, Parulkar A, Singh R, Kadam R, et al. Field sales force model to increase adoption of a novel tuberculosis diagnostic test among private providers: evidence from India. BMJ Glob Health. 2020;5(12):e003600. doi: 10.1136/bmjgh-2020-003600 33376100 PMC7778745

[34] Kuupiel D, Bawontuo V, Drain PK, Gwala N, Mashamba-Thompson TP. Supply chain management and accessibility to point-of-care testing in resource-limited settings: a systematic scoping review. BMC Health Serv Res. 2019;19(1):519. doi: 10.1186/s12913-019-4351-3 31340833 PMC6657084

[35] Bui T. Vietnam’s COVID-19 testing scandal goes viral. East Asia Forum. 2022. [cited 2024 Jan 12] Available from: https://www.eastasiaforum.org/2022/01/21/vietnams-covid-19-testing-scandal-goes-viral/.

