# Supplementary material for: Leveraging a digital network of pharmacy professionals to test a technology-assisted model to improve pharmacy access to quality-assured COVID-19 rapid antigen tests approved for self-use in Vietnam
Source: PLoS One. 2025 Mar 19;20(3):e0318331. doi: 10.1371/journal.pone.0318331 (PMC11922224; doi:10.1371/journal.pone.0318331)
Supplement: S1 File — (PDF) [file pone.0318331.s001.pdf]

SwipeRx Viet Nam: Understanding Access to Quality-Assured COVID-19 Ag-RDTs for Self-Testing Survey

Consent Form

SwipeRx is conducting research about understanding access to quality assured COVID-19 rapid antigen tests (Ag-RDTs) including tests approved for self-use in Viet Nam.

You are being invited to participate in this survey because you are (1) working in a pharmacy as either a pharmacist, pharmacy assistant, pharmacy manager, or a pharmacy owner, (2) provided consent to be contacted again in the March 2022 baseline survey, or (3) have been coached and trained by SwipeRx during activation phase. If you agree to participate, we will ask you questions about supply chain and your capacity to help clients provide COVID-19 testing kits. The survey will take approximately 10-15 minutes to finish.

All data is stored securely, and we guarantee your confidentiality. To protect your privacy, this survey does not ask for any identifiable information except mobile number and pharmacy name. Only overall, aggregated results will be used when sharing findings with stakeholders. The results of this survey will not be used for any commercial purposes.

Your participation in this survey is voluntary. You are free to decline to participate or to leave the survey at any time. If you decide not to participate in the survey, this will not affect your access to, or services provided by SwipeRx. If you participate in this survey, you are free not to answer questions that make you uncomfortable or that you do not want to answer. By starting the survey, you agree to participate in the study and have accepted the terms and conditions. After you complete the survey, you will receive 150.000 VND on your mobile phone as token of appreciation for your time completing the survey. Your mobile phone information will not be linked in any way to the responses you provide in the survey.

Terms and Conditions

1. This survey will be accessible through SwipeRx starting **<date>** and will remain accessible on the application until the full sample is complete.
2. This survey is carried out entirely within the *SwipeRx* mobile phone app.
3. To be eligible to complete the survey you must:
    a. Be registered as a *SwipeRx* user.
    b. Be at least 18 years old
    c. Be a pharmacy professional (non-student)
    d. Work in a retail pharmacy (only 1 respondent per pharmacy)
4. Participation in this survey is free.
5. You will receive 150.000 VND credit as a token of appreciation.
6. Each participant can only complete the survey once.
7. Credit transfer will be processed a week after you complete the survey.

8. Credit transfer is provided given that you answered at least 80% of the questions.
9. Credit transfer is provided directly to the mobile number you provide in the field below. If you have not received the credit after two weeks of taking the survey, please contact hello@swiperxapp.com.

*Q1. Do you agree to participate in this survey?

- I agree to take part in the survey
- I do NOT agree to take part in the survey ***[Redirect to survey end page if selected]***

*Q2A. Which mobile number would you like to use to receive the token of appreciation for taking this survey (given requirements are met, according to the terms and conditions above)?

- *(free text)*

Q2B Please enter the 4-digit code provided by your SwipeRx agent or co-worker in the box below. (If you did not receive "SwipeRx")

*Q3. What is the name of the pharmacy you work in?

- *(free text)*

*Q4. What is your *main* pharmacy professional role? *(select one)*

1. Pharmacist
2. Pharmacy assistant
3. Pharmacy owner
4. Pharmacy manager
5. Other: *[open text]*

[End survey if D4 = 5]

*Q5. How old are you?

- *[numbers 18 - 100]*

[End survey if Q5 < 18]

*Q6. Are you working in a retail pharmacy?

1. Yes
2. No

[End survey if Q6 equal to 2]

*Q7. Are you currently stocking at least one COVID-19 Ag-RDT (any type)?

1. Yes
2. No

Demographics

*D1. What province do you currently work in?

(drop down: all provinces)

*D2. Which district do you currently work in?

(drop down: all districts)

*D3. What is your gender?

1. Woman
2. Man
3. Other/prefer not to say

Awareness

AW1. What is the difference between COVID-19 Ag-RDT approved for self-use vs COVID-19 Ag-RDT approved for professional use?

1. The person performing a self-use test is not a trained healthcare professional
2. The self-use tests packaging has detailed Instructions for Use (IFUs) for the user
3. **1 and 2****
4. None of the above
5. Don't know

*

Client Profile and Volume

CV1. Approximately how many pharmacy clients visit your pharmacy in a typical day?

- *[numbers 0 – 500]*

CV3. In a typical week, which types of client demographics purchased Ag-RDTs (any type) most? [select all that apply]

a) Health Workers
b) Senior Citizens
c) Business owners
d) Individual clients with COVID-19 symptoms
e) Others, please specify: [open text]

[CV3 will appear if CV2 != 0]

CV4. How often client purchase Ag-RDTs(any type)?

1. Less than once every other month
2. Once every other month
3. Between 1-3 times a month
4. Once a week
5. More than once a week

CV5. What are some questions that clients ask you related to COVID-19 Ag-RDTs? [open text]

Knowledge and Training

*KT1. Have you received training or education on COVID-19 related care or products within the last 1 year? If so, please indicate the topics [select all that apply]

a) COVID-19 symptoms and risk factors
b) PCR testing
c) Ag-RDTs approved for self-testing
d) Ag-RDTs approved for professional use
e) Rapid antibody testing
f) COVID-19 variants
g) Others, please specify [open text]
h) None of the above [make answer exclusive]

KT2. Who provided the COVID-19 related training? [select all that apply]

a) DOH
b) Distributor
c) Manufacturer
d) SwipeRx
e) Others, please specify [open text]

[KT2 will appear if KT1 is not equal to g]

Product Purchasing, Stocking, and Other Practices Section

SCP1_A. How many suppliers do you source COVID-19 testing kits from currently?

[number: 0 - 30 suppliers]

[SCP1_A will appear if Q7 equal to 1]

*SCP1B. Please select the type of suppliers you source COVID-19 testing kits from (select all that applies)

a. Wholesaler
b. Distributor
c. Manufacturer
d. Others, please specify: (open text)

*SCP2. On average, how often do you make purchases from your suppliers for COVID-19 testing kits?

1. Less than once every other month
2. Once every other month
3. Between 1-3 times a month
4. Once a week
5. More than once a week

[SCP2 will appear if Q7 equal to 1]

*SCP3. How do you buy the COVID-19 testing kits from your suppliers? [select all that apply]

a) Purchase from salespeople who visit the pharmacy
b) Purchase online
c) Purchase by phone
d) Physically visit wholesales or distributor to purchase
e) Others, please specify: [open text]

[SCP3 will appear if Q7 equal to 1]

*SCP4_A. For all COVID-19 Ag-RDTs currently stocked at your pharmacy, please provide a photo of the product with brand name and packaging size readable. (photo capture)

*SCP4_B. Please provide the following information for each test currently available in your pharmacy captured in your photo (please indicate 0 for other tests not available)

	Brand Name	Type (Nasal or NP) (choose one)	# of box stocked	# Tests per box/SKU size (e.g box of 1,4,5,10,20,25)	Price per box paid by pharmacy (in VND)	Average # of Tests Sold Weekly
Test 1						
Test 2						
Test 3						
Test 4						
Test 5						

*SCP4_C. What is the most popular brand of COVID-19 Ag-RDTs these days? (open text)

*SCP5. What convinced you to stock the brands/tests you currently stock? [Select Top 3]

a) Purchase Price
b) Consumer Preference
c) Discounts or other deals from supplier
d) Place of manufacturer
e) Global approvals
f) Information provided through SwipeRx app newsfeed
g) Training provided by SwipeRx
h) Information provided through SwipeRx Facebook
i) Local approvals
j) Others, please specify: [open text]
k) None of the above [make answer exclusive]

SCP6. What are the main factors influencing sales of COVID-19 tests in the last 1 month? (select all that apply]

a) Price
b) Consumer Demand
c) Discounts or other deals
d) Place of manufacturer
e) Global approvals
f) Local approvals
g) COVID-19 surge
h) Others, please specify [open text]
i) None of the above [make answer exclusive]

[SCP6 will appear if D7 equal to 1]

SCP9_A. In the past month, how many times did you experience stock out of approved COVID-19 testing kits (any type)? [number 0 - 50]
[SCP9_A will appear if D7 equal to 1]

SCP9_B. How fast do you receive stocks of COVID-19 testing kits after ordering it from supplier?

1. less than 4 hours
2. 4 - 12 hours
3. half day to 1 day
4. 1 - 2 days
5. more than 2 days

*SCP10. Which of the following activities related to COVID-19 do you currently participate in? [select all that apply]

a) In-pharmacy COVID-19 testing
b) Referring clients for COVID-19 to a health facility
c) Counselling clients about COVID-19
d) Other, please specify: [open text]
e) None of the above: [make answer exclusive]

*SCP10_A. Please indicate the brand of test you used for in-pharmacy COVID-19 testing: [open text]
[SCP10_A will appear if SCP10 equal to "a"]

SCP11. Which factors relate to "quality" of Ag-RDT in your opinion? [select all that apply]
a) Manufacturer Location
b) Global approvals
c) Local approvals
d) Packaging
e) Price
f) Others, please specify: [open text]
g) None of the above [make answer exclusive]

Attitudes

A1. I think pharmacy professionals have a role to play in counselling clients to correctly self-test for COVID-19 and refer for further care to a health facility if needed

1. Strongly disagree
2. Disagree
3. Agree
4. Strongly Agree

A2. How confident are you in your ability to counsel clients about COVID-19?

1. Not Very Confident
2. Slightly confident
3. Confident
4. Very confident

[A2 will appear if SCP10 = "c"]

Opportunities and Barriers Section

OB2. Which of the following would increase pharmacy sales of quality assured self-use Ag-RDTs ie Flowflex or Humasis nasal 5-test packs? (select all that apply)

a) Digital Training or education
b) In-pharmacy material
c) Visible merchandise of COVID-19 Ag-RDTs on pharmacy countertops
d) Reduced pricing
e) Promotion from MoH
f) Others, please specify: [open text]
g) None of the above [make answer exclusive]

OB3. What type of support would motivate you to provide more quality assured COVID-19 self-tests at your pharmacy? (select all that apply)

a. Discounts on quality assured tests
b. Credit when purchasing quality assured tests
c. Free quality assured tests (product promos)
d. Pharmacy equipment
e. Training
f. Recognition from MOH or Pharmacy association
g. Others, please specify: [open text]
h. No incentives would motivate me [make answer exclusive]

F1. Would you be interested in participating in future programs to improve community awareness and access to quality assured COVID-19 Ag-RDT for self-use through your pharmacy in future?

1. Yes
2. No

F2. Do you consent for SwipeRx to contact you? If so, please provide the best ways to contact you

1. No, I do not consent

2. Yes, I consent. My contact information is: *[open text]*

[F2 will appear if F1 = "Yes"]
